# How to Make the Hospital an Option Again: Midwives’ and Obstetricians’ Experiences with a Designated Clinic for Women Who Request Different Care than Recommended in the Guidelines

**DOI:** 10.3390/ijerph182111627

**Published:** 2021-11-05

**Authors:** Floor Opdam, Jeroen van Dillen, Marieke de Vries, Martine Hollander

**Affiliations:** 1 Department of Midwifery, The University of Midwifery Education & Studies, Universiteitssingel 60, 6229 ER Maastricht, The Netherlands; 2 Department of Obstetrics and Gynaecology, Amalia Children’s Hospital, Radboud University Medical Centre, Geert Grooteplein Zuid 10, 6525 GA Nijmegen, The Netherlands; Jeroen.vanDillen1@radboudumc.nl (J.v.D.); Martine.Hollander@radboudumc.nl (M.H.); 3 Institute for Computing and Information Sciences, Radboud University, Toernooiveld 212, 6525 EC Nijmegen, The Netherlands; marieke.devries@ru.nl

**Keywords:** maternity care, personalised care, woman-centred care, high risk, care outside the guidelines, treatment refusal, homebirth, shared decision making, autonomy, The Netherlands

## Abstract

Background: An increasing number of maternity care providers encounter pregnant women who request less care than recommended. A designated outpatient clinic for women who request less care than recommended was set up in Nijmegen, the Netherlands. The clinic’s aim is to ensure that women make well-informed choices and arrive at a care plan that is acceptable to all parties. The aim of this study is to make the clinic’s approach explicit by examining care providers’ experiences who work with or within the clinic. Methods: qualitative analysis of in-depth interviews with Dutch midwives (*n* = 6) and obstetricians (*n* = 4) on their experiences with the outpatient clinic “Maternity Care Outside the Guidelines” in Nijmegen, the Netherlands. Results: Four main themes were identified: (1) ”Trusting mothers, childbirth and colleagues”; (2) “A supportive communication style”; (3) “Continuity of carer”; (4) “Willingness to reconsider responsibility and risk”. One overarching theme emerged from the data, which was “Guaranteeing women’s autonomy”. Mutual trust is a prerequisite for a constructive dialogue about birth plans and can be built and maintained more easily when there is continuity of carer during pregnancy and birth. Discussing birth plans at the clinic was believed to be successful because the care providers listen to women, take them seriously, show empathy and respect their right to refuse care. A change in vision on responsibility and risk is needed to overcome barriers such as providers’ fear of adverse outcomes. Taking a more flexible approach towards care outside the guidelines demands courage but is necessary to guarantee women’s autonomy. Key conclusions and implications for practice: In order to fulfil women’s needs and to prevent negative choices, care providers should care for women with trust, respect for autonomy, and provide freedom of choice and continuity. Care providers should reflect on and discuss why they are reluctant to support women’s wishes that go against their personal values. The structured approach used at this clinic could be helpful to maternity care providers in other contexts, to make them feel less vulnerable when working outside the guidelines.

## 1. Introduction

An increasing number of maternity care providers—particularly in high-income countries—encounter highly-educated and well-informed pregnant women who request less care than recommended by medical professionals [1,2,3,4,5,6,7,8,9,10,11,12,13,14,15]. These women desire maternity care that is more in line with their personal ideas and preferences instead of merely sticking to what medical guidelines recommend [1,2,4,5,7,10,11]. They strive for autonomy, personal choices. Their choices are not always against medical guidelines, but they can also be against local customs. Examples of requests outside the guidelines are: birthing without skilled attendance (freebirth, unassisted childbirth), birthing at home in a country where home birth is not well integrated into the maternity care system, declining induction of labour when indicated or declining foetal monitoring during labour [2,4,11,16,17]. Examples of requests against local customs are: a vaginal breech birth ‘on all fours’ or giving birth in a vertical position. 

In the Netherlands, home birth is well-integrated in the maternity care system. Women with a low-risk pregnancy in the Netherlands receive care from a community midwife. These women can choose to birth at home, or midwife-led in a hospital. Women with a high-risk pregnancy are referred to the hospital, where they receive obstetrician-led care Midwives in the Netherlands either work as community midwives in primary care or in the hospital under supervision of an obstetrician. Community midwives typically work in group practices where women receive care from several midwives. However, some community midwives work with a caseload model of care, in which their clients receive care from one known midwife throughout their pregnancy, birth and the early postpartum period [18,19]. 

Motivations of these women can be classified in different themes: an instinctive trust in pregnancy and childbirth, a critical approach towards medical interventions and an experienced lack of control and choice during their previous birth experience, which can be a traumatic experience [1,7,10,11,15]. In the latter case, women then make plans in their next pregnancy which they feel protect themselves against a repeated experience of loss of autonomy and control [1,7,10,11,15]. 

The working relationship between a woman and a care provider can be threatened when a woman requests less care than recommended. These requests can cause care providers to feel uncomfortable, vulnerable, fearful and frustrated [3,4,8,13]. Internationally, steps have been taken to support maternity care providers in caring for women who request less care than recommended. Several professional organisations have issued guidelines on this topic. These guidelines stress the importance of providing sufficient information so that women can make informed decisions, practicing shared decision making and respecting maternal autonomy [14,20]. In addition, in the Netherlands and in the UK, designated outpatient clinics for maternity care outside the guidelines were established [17,21,22]. 

In the Netherlands, the university hospital in Nijmegen has started an outpatient clinic “Maternity Care Outside the Guidelines”. The clinic is characterised by its multidisciplinary approach and the large amount of time taken for a consultation, spread over a minimum of three structured visits. Women can be referred to the clinic, either by a hospital-based midwife, community midwife or by an obstetrician. The clinic’s aim is to have a constructive and respectful dialogue about women’s preferences, and the risks, benefits and alternatives of a care plan. Shared decision making (SDM) is one of the fundamental principles of the designated clinic. Since the start of this clinic in 2015, we see approximately five women per year. Van der Garde et al. [17] assessed the outcomes of the clinic’s first three years. There were no serious adverse maternal or perinatal outcomes. 

Holten et al. [23] examined ten cases of women who chose a home birth in a high-risk pregnancy (breech, twins, previous CS), with the aim of exploring how this wish was negotiated during consultations.

They found a pattern in women’s experiences: nine women initially planned to give birth in the hospital, but eventually decided to birth at home, because of an experienced conflict with their care provider during negotiation of their birth plan. During those conversations, women encountered inflexible care providers who were not willing to consider their birth plans, nor were they willing to make any concessions. Most importantly, women experienced a lack of autonomy. These experiences resulted in the decision to leave the regular maternity care system: a ‘negative choice’. A negative choice can be defined as a decision made *against* something, rather than *for something*. In contrast to the cases studied by Holten et al. [23], women who visited the designated clinic described in the current study did not make negative choices. For many, the hospital had become an option again. Some women still chose to birth at home after visiting the clinic, but they did so because they felt it was the best option for them, after weighing all the risks, not because the hospital left them no choice. We decided to study care providers’ experiences with the outpatient clinic “Maternity Care Outside the Guidelines” at the University Hospital in Nijmegen, the Netherlands. The aim of this study was to elucidate what providers at the designated clinic do differently from those in the study by Holten et al. [23], in order to prevent women from making negative choices. 

## 2. Materials and Methods

The consolidated criteria for reporting qualitative research (COREQ) [24] were used to describe data collection and data analysis. 

### 2.1. Research Team and Reflexivity 

Care providers were interviewed by F.O., She is a fourth-year student midwife at the Midwifery faculty of Zuyd University in Maastricht who had no prior experience in conducting interviews. None of the participants were known to the interviewers prior to the interviews. M.H. and J.v.D. are both obstetricians, who work together in the designated clinic. M.d.V. is a medical decision psychologist with an interest in shared decision making and birth trauma. All authors believe in the importance of the clinic, which may have influenced the content of this article.

### 2.2. Study Design 

This single-centre qualitative study was carried out between August 2019 and June 2020 at the Radboud University Medical Centre in Nijmegen, the Netherlands. The care providers involved in ten cases from the designated clinic were interviewed in order to obtain insight in their experiences with the clinic, their interactions with the women involved and the decision-making process which led to the final birth plan. Interviews with the women and their partners were carried out simultaneously but will be reported in a different article.

### 2.3. Study Population

Participants were a purposive sample of community- and hospital-based midwives and obstetricians involved in ten selected cases from the designated clinic (see Table S1 in Supplementary Materials). These ten specific patient cases were chosen because they are a good representation of the requests encountered at this clinic. The ten women involved agreed to the study and were also interviewed about their experiences, which we will report on later in a separate article. 

All interviewed midwives and two of the obstetricians work outside of the designated clinic, which is run by the other two interviewed obstetricians. The other participants referred one or more clients to the clinic and/or were involved during the birth of a woman who requested less care than recommended. Most participants have been involved with several cases since the start of the clinic. However, in the interviews only one specific case was discussed. Contact details of the participants in the current study were known to the researchers through the clinic. All participants were approached by either e-mail or telephone.

### 2.4. Data Collection

Data were collected through in-depth semi-structured interviews performed between August 2019 and June 2020, after verbal and written consent was obtained. Prior to the interviews, there had been e-mail contact with all participants to explain the aim and methods of the study and issues regarding confidentiality. Eight interviews occurred face-to-face and were performed at the participants’ workplace. Two interviews were conducted over Skype, due to the COVID-19 pandemic. A topic list was used to guarantee consistency between the interviews. The topics addressed in the interviews are shown in Table S2. Some topics were added later as new (sub-)themes arose from prior interviews. Interviews lasted between 30 and 90 min and were audio-recorded and transcribed verbatim. Ethical approval was obtained from the medical ethics committee of the Radboudumc in February 2019. All transcripts were anonymised. The audio recordings and transcripts were encrypted and stored anonymously in a password protected university storage system. Quotes were translated from Dutch into English by the first author. The collection of data stopped after 10 interviews. Data saturation was reached after 8 interviews. 

### 2.5. Data Analysis 

Once transcribed, the transcripts were read through and notes were taken to become familiar with the data. Subsequently, the transcripts were coded using the qualitative data analysis software Atlas.ti (ATLAS.ti Scientific Software Development GmbH, Berlin, Germany). The initial open coding was followed by axial coding and selective coding. During the coding process memos were written to engage with the data, to clarify codes and to determine the relations between different codes and themes, which facilitated the axial and selective coding process. To safeguard intercoder reliability, several transcripts were coded independently by two researchers and any discrepancies were discussed. Four core categories were found, which were discussed within the research team until consensus was reached. 

## 3. Results

Ten care providers were interviewed: four obstetricians, three community midwives, two caseload midwives and one hospital-based midwife. Table S1 (Supplementary Materials) illustrates the characteristics of the participants, their role in the care process and their client’s pregnancy characteristics. At the designated clinic, requests for care outside the guidelines are very diverse [17]. However, the most common request in this study for women who visited the clinic was to be cared for by their own known (community) midwife, instead of obstetrician-led care, during a vaginal birth after a previous caesarean section, hereafter abbreviated as VBAC. Often, after negotiation at the clinic, a care plan closer to the protocol than the women’s initial request is agreed upon. However, in these ten cases, there was no substantial deviation from the initial “outside of protocol” birth plan to a more standard birth plan during intrapartum care. 

Nonetheless, in several cases, women were finally willing to accept certain (emergency) interventions, while some of those interventions—as described in the original care plan—were initially not acceptable to them. For instance, in the case of obstetrician 1, the patient initially refused an ultrasound scan during the intertwin delivery interval, but changed her mind after negotiations in the clinic. 

This patient also refused IV-access during birth and administration of oxytocin postpartum, but accepted both when she started haemorrhaging after birth. 

Four main themes were identified: “Trusting mothers, childbirth and colleagues”, “A supportive communication style”, “Continuity of carer” and “Willingness to reconsider responsibility and risk”. One overarching theme was found: ‘Guaranteeing women’s autonomy’. Autonomy comprises the main reason why tailoring care is important for the interviewed care providers. 

### 3.1. Trusting Mothers, Childbirth and Colleagues 

To the participants, trust is essential in the working relationship between woman and care provider. Participants perceived that when women have had a negative birth experience in the past, there is a pre-existing lack of trust. This placed them in a difficult position.

*“People are often distrustful when they enter my room. Because they went through something and don’t just give in. They are often resistant, like ‘I really know what I want this time, what I don’t want and I’m going to fight for it, fight against you. I need to fight because you’re going to try to convince me to consent to recommended care.’ So that’s how the first conversation starts. At that point you’re already a few steps behind.”*

(Obstetrician 1)

This obstetrician also describes how trust between women and care providers at the outpatient clinic prevents women from making negative choices:

*“As long as I can monitor and determine whether intervention is necessary. […] So, when people allow me to monitor, if I can monitor mother and baby, I’m fine with it. […] It hasn’t happened to me yet that I wasn’t allowed to intervene while intervening was crucial. I don’t know why. Maybe, like we discussed before, that I build trust from the beginning on and that people think ‘She will only intervene when it is really necessary’.”*

(Hospital midwife 1)

Care providers felt that they needed to invest in mutual trust before they could effectively offer care. Seeing the woman as a person, and showing motivation to make a woman’s birth experience a positive one are mentioned examples of ways to build on trust. According to the participants, speaking about medical risks during the first consultation, could be seen by women as ‘closing the door’ and would confirm their distrust in care providers:

*“I know caregivers who say: ‘You need to trust me, because I want what’s best for you’. I think that trust is something you need to earn so you need to listen to your patient. Not until she asks about your idea of the situation, only then you will tell her. The chance of her listening to you is so much better than when you immediately start with: ‘I think this is right for you’.”*

(Caseload midwife 1)

Taking time for consultations is also noted to be crucial. In summary, building trust is all about investing time, showing interest in the person behind the request, and not rushing in with information on risks.

*“Trust is built by investing time, the first consultation at the clinic lasts an hour. During this consultation I want to know ‘who is this woman?’ […] It’s not about medical questions, I just want to get an impression of this person. […] Asking her about her vision is important, even if it differs from mine. […] That’s how I start my conversations at the designated clinic.”*

(Obstetrician 2)

Other obstetricians mentioned that in their work context there is insufficient time to build a relationship of trust. The duration of regular consultations—outside the designated clinic—is too short to really get to know a woman. In the hospital, there is no continuity of carer which makes building a trusting relationship more challenging.

*“I would prefer that the care provider who did the whole care process at the clinic, would also attend the birth. And yes, that is logistically difficult and I understand it causes a certain pressure. It would be ideal for me, to have that continuity. Because I think it is sometimes inconvenient to perform an invasive medical procedure, when the woman does not know or trust you yet. For these procedures, a trusting relationship is essential. And you can’t always achieve that in ten minutes.”*

(Obstetrician 4)

Participants remarked that trusting your client is of great importance in an emergency situation. You need to trust that your client will not refuse a life-saving intervention, especially if she agreed to life-saving interventions earlier on. One midwife said she trusted her client, because she was perfectly informed and conscious of her decision:

*“I trusted her because she could assess risks better than I could. Well, she just immersed herself really well into this specific medical topic. […] Besides, she was just really good in her reasoning, which made me have faith in a good outcome. […] I was not afraid that I would be traumatised myself because I had to conform to her choices. No, I trusted her that if I needed to do a certain intervention, for example give an oxytocin injection, that she would allow me to.”*

(Community midwife 1)

Care providers reported that for some women, this permission to act in an emergency situation, is exclusively given to the care providers they know and trust, and not to the entire medical team.

*“She trusted that if I wanted to intervene, I would have a good reason for it. However, trusting me did not mean that she trusted the entire team. So, the difficult thing was that I couldn’t just leave her with anyone. Moreover, I could foresee that some of my colleagues would have a lot of difficulty with her birth plan. I was not sure that her wishes would be respected.”*

(Obstetrician 1)

Several care providers emphasised that trust in physiological childbirth motivates them to personalise care, even if this involves crossing guidelines. They declared that allowing childbirth to happen without interventions was easier when they trusted women’s intuition and instinctual knowledge.

*“There was no need to intervene in the process because there was continuous foetal monitoring and she (the client) was handling the contractions really well. In the end she had the urge to push […] and her partner said ‘I feel that the baby is coming closer’. I thought: that’s fine, I don’t need to go and check. […] The baby was doing well so I didn’t need to intervene. All that time I sat on my chair and observed.”*

(Hospital midwife 1)

Community midwives as well as obstetricians experienced that a multidisciplinary approach in the clinic led to less feelings of vulnerability and a feeling of trust and shared responsibility. Conversely, several community midwives called for more trust in them as providers and their professional expertise from other obstetricians and hospital-based midwives (not involved in the clinic):

*“It should be more common. That the collaboration is good and pleasant, that the obstetricians trust the community midwives. That we make sensible decisions and really don’t do crazy things.”*

(Community midwife 1)

### 3.2. A Supportive Communication Style

For the participants, a supportive communication is crucial in order to build trust and personalise care. A supportive style involves active listening, showing genuine interest, empathy, respect and attention. Exploring and clarifying women’s preferences may give a better understanding of the context within which the woman is making certain choices, that initially might seem irresponsible from the caregiver’s own perspective.

*“Well, it helps if I understand, so I ask questions so that I can find out why it is so important to… I always try to find the motive behind the request, although some people are very obvious in formulating their question. Whereas other people say: ‘I want to birth at home’, while they actually mean: ‘I had a bad experience in a hospital and I feel like I had no say over my own body and I hope I will have that with a midwife.’”*

(Caseload midwife 1)

Additionally, both obstetricians and midwives noted that understanding the woman as a person and seeing things from her perspective helped them in the decision-making process. Care providers attached much value to transparency. They pointed out that being honest about what may happen during birth, involving women in clinical decisions and expressing your personal boundaries are fundamental.

*“I think what happens often is that there is non-verbal communication, but no verbal communication. Women see the disturbed faces of care providers, but they are not told what’s going on. This can do a lot of harm […] when care providers are afraid to say what’s going on during birth.”*

(Community midwife 1)

As mentioned before, the aim of the clinic is to support women in making informed decisions. Midwives and obstetricians were asked about their opinions of SDM. Almost every participant stated that SDM contributes to personalised care. The information discussed about medical risks and proposed interventions ought to be complete and realistic. Women should feel encouraged to make their own decisions and receive advice while making them.

*“I see myself as a guide. To provide information, and not just present it to her and say: ‘OK, this is it and make your decision.’ I think supporting her in making decisions is important. For example, I say: ‘You’re choosing this, but looking at what I know of you, this choice doesn’t seem to fit you altogether. Is this really the right choice for you? Does it fit your way of thinking and living, because to me it doesn’t match.’”*

(Obstetrician 1)

Despite the obstetrician’s support for SDM, she doubted whether a true ‘shared’ decision is always realistic.

*“Yes… what is a shared decision? A real shared decision does not exist of course. Because, imagine someone is pregnant and she does not want the baby and the partner does not agree, or the other way around, you can’t keep half of the baby. You keep it or you don’t keep it. It’s a ‘yes’ or a ‘no’. So, in the end there’s one person who decides. And that is the one who can say ‘yes’ or ‘no’. And that’s her (the woman).”*

(Obstetrician 1)

### 3.3. Continuity of Carer

The interviews revealed that time and continuity allowed women and care providers to build a bond of trust. Once there is continuity of carer, care providers get to know their clients and are better able to adjust their care to the personal needs of the women. Continuity of carer was perceived to be the best way towards more personalised care.

*“I think for many pregnant women continuity of carer is important. I think there are some women who find it less important, but if you know each other well it’s easier to sense what the woman needs during birth. (…) Knowing each other helps… to act better if you get into a situation. So, I think it contributes to a healthy birth. […] So, I think it really contributes to good outcomes, for mother and child.”*

(Community midwife 1)

For the interviewed caseload midwives, trust was the main reason why they chose to work as a caseload midwife.

*“In the end, giving birth is about that people can shut their minds down and let their bodies do the work. And we, modern people are taught to think a lot and we are less used to listening to our body, our belly. Mutual trust can help women to trust their bodies, that they feel like they are able to do it on their own. And yes, you can give them some tools for that and one of these tools is continuity of carer.”*

(Caseload midwife 1)

Caseload midwives mentioned that working with a continuity of care model led to more job satisfaction, but they experienced that this is not always financially viable.

The majority of the participants stressed that there is a lack of continuity within the regular maternity care system. Community midwives expressed their preference for a maternity care system in which they are also qualified to attend births that are normally attended by obstetricians, such as a vaginal birth after a caesarean section (VBAC). Midwives feel confident and capable in broadening their area of expertise. They aspire to share care with obstetricians in order to make continuity of carer possible.

*“A vaginal birth after a caesarean with continuous electronic foetal monitoring attended by a community midwife… that’s fine. I think there are more indications […] that I would feel capable to be responsible for. Such as meconium-stained liquor or […] attending births of GBS-positive women who receive intravenous antibiotic prophylaxis. I completely support that.”*

(Caseload midwife 2)

In contrast to the other participants, one midwife deemed continuity of carer not vital. For her, continuity of the care plan is what is important in order to personalise and optimise maternity care.

*“Well, I think, I don’t think you necessarily need to have one care provider in order to achieve personalised care, because we also don’t do that in our practice. […] So, we (midwives in the practice) […] have weekly meetings, and then for example, the client we talked about now, is discussed. Like ‘what is her wish’ and then everyone is informed. So, I don’t think continuity (of carer) is necessary.”*

(Community midwife 2)

### 3.4. Willingness to Reconsider Responsibility and Risk

Participants consistently mentioned that guidelines should be handled more flexibly, and care plans should be adjusted to women’s personal needs and preferences. Caring for women who request less care than recommended gives participants fulfilment. Moreover, they noted that it made their jobs and relationships with women more meaningful.

*“Things would be easy […] if women would completely conform […]. It makes everything more complex and beautiful that women make their own decisions. Being able to make your own decisions as a woman is really important. I think it’s a good development because giving birth is a rather intense life-event, and that’s something you should have complete control over. And with good information you can make choices that fit you. I don’t think that every woman fits in the same protocol.”*

(Community midwife 1)

Care providers were asked whether they feel responsible for the consequences of a woman’s choice. Each participant pointed out that, for them, being responsible means taking a proactive role in ensuring that women are well-informed.

*“My main goal in a consultation is that patients make a well-considered choice when they have all the available information […] And that we finally arrive at a care plan that she supports, regardless of the outcome, that she doesn’t regret afterwards. […] Whether that’s inside or outside the protocol. […] And if the outcome turns out to be bad, she’s able to say: ‘It is what it is because I chose this consciously’.”*

(Obstetrician 1)

All interviewed care providers declared to support the clinic’s approach. However, there are some differences between care providers. There is a difference in the extent in which care providers are willing to go along with their client’s wishes. In other words, there is a difference in the extent in which they are willing to accept the risks that come along with their clients well-considered decision(s). Two participants explicitly stated that they do not feel responsible for the consequences of decisions made by a well-informed client. Whereas three other participants—working closely with but not for the designated clinic—explicitly stated that they think it is important to take a woman’s request seriously, but that this does not necessarily mean that they would agree to a request that is in conflict with their own values. Reasons mentioned were a fear of adverse events, a fear to become traumatised when these events occur or a fear of legal consequences. The other participants did not make their boundaries as explicit as the beforementioned participants.

*“I think it’s important to fulfil my medical responsibilities… Yes, I don’t want to cross my own limits. So, when someone has certain wishes and I am medically responsible for this birth, I can be held accountable afterwards. For instance, by a disciplinary judge. […] No continuous electronic foetal monitoring is no option for me. And often, if you discuss that with people, they are fine with it. They appreciate that you are willing to look at their wishes and see to which extent they are possible.”*

(Obstetrician 4)

In the last several years, maternity care providers have experienced an increasing tendency to protect themselves against legal repercussions. Because of this, participants document their care more scrupulously than they used to do.

*“Well, of course, as a result of all these guidelines and—if I look at 27 years ago when I started as a midwife… back then, women with meconium-stained liquor just birthed at home. I was less anxious than I am now—if you want to call it anxious—I think I’m more careful these days. It has something to do with guidelines and perinatal audits where you need to justify your actions.”*

(Community midwife 3)

Understanding and seeing the client as a person are reasons why participants decided to agree to a woman’s request. Additionally, valuing a positive birth experience as an outcome in its own right enabled participants to better understand women’s preferences.

*“I believe in freedom of choice. […] And to have a pregnancy outcome… I don’t just look at the medical outcomes, the woman’s birth experience is just as important. In my eyes, a birth experience is positive when there’s trust and freedom of choice.”*

(Obstetrician 2)

Work experience and personal growth enabled care providers to personalise care more easily.

*“I’ve been a midwife for nine years now and I’ve grown as a person. If I had to do this while I was recently graduated, I may not have done this, because I would have been afraid of… trouble, I think. Now I’m able to… I understand… I can empathise better with clients.”*

(Community midwife 2)

Despite the participants’ overall positive attitude towards personalised care, some disclosed that caring for women who request less care than recommended can be demanding. A major challenge is weighing and taking risks when working outside the guidelines.

*“Medically… I don’t always think it’s sensible. But if you look at absolute risks […] and you say: ‘I had a previous caesarean and I really want to birth at home with my midwife’, then the risk of something dramatic is really small. The chances things turn out well are really big. Weighing risks is difficult. […] I don’t know what my risk is of me getting involved in a car crash or a plane crash or die during childbirth. So, what does this 0.5 percent risk tell me? I have no idea.”*

(Obstetrician 3)

The second challenge is disagreement with other professionals about case management. Community midwives feel vulnerable when more apprehensive colleagues deem their actions irresponsible when they are willing to assist a woman who wants to give birth outside the guidelines. Sometimes the care provider on duty does not feel comfortable with the birth plan agreed on at the clinic, which leads to discussion during birth. In those cases, a different care provider who does support the birth plan is asked to take over. 

Participants surmised that some of their colleagues hesitate to work outside the guidelines because every health care professional has a personal perception of risk and weighs risks differently. Therefore, reaching agreement on a birth plan with colleagues can be time-consuming:

*“Sometimes it’s complicated to discuss everything with five midwives. We’ve talked this case through many times. ‘What’s your personal limit?’ ‘What’s yours?’ ‘How do we do this then?’ ‘What do you accept?’ ‘What not?’ ‘What do we do when a woman with a previous caesarean needs continuous support when at the same time there is a homebirth?’ All that kind of stuff. Well, it’s inconvenient sometimes.”*

(Community midwife 3)

Since the start of the clinic, most consultations have led to care plans acceptable to all parties. Participants remarked that all this experience, however, has not yet led to new protocols. They call for a decision-making format in order to stop having to discuss something elaborately that has been discussed many times before.

*“You know, it costs a lot of time. I doubt if that is necessary. […] Because you want something that’s different from the guideline. And to which extent can I, as a midwife, care for this woman? […] I think, if you’ve said ten times ‘previous caesarean section, this woman wants to birth in the hospital with her own community midwife, the foetal heart pattern is interpreted by us (medical personnel) and the woman gets intravenous access’. Well, then you can say after five times, it went well so this is our new policy.”*

(Community midwife 3)

### 3.5. Core Theme: Guaranteeing Women’s Autonomy

The concept that all themes share is guaranteeing women’s autonomy. Participants explained that trust is regained and maintained by respecting and guaranteeing women’s autonomy, and good communication involves supporting a woman in making choices, but leaves the final decision to her. Every woman should be in control over her own health, her baby’s health and the care she accepts or declines.

One midwife highlighted that her key motivation to work outside the guidelines is her commitment to women’s autonomy.

*“It is important that women make their own decisions, because it’s their own body and their own child. I don’t think that it only involves pregnancy, birth and the puerperium, but I think as long as you have children, you are the one to decide how to raise them and how you manage your health and theirs.”*

(Community midwife 1)

Another midwife describes how personalised care enables women to be actively involved in their care:

*“Personalised care is about giving time and attention. […] Really empowering women. […] I think it’s very important that people feel empowered and think about things… how birth can turn out. I always say: ‘Even if it ends in a caesarean section, you can end up feeling good about it when you felt involved in decisions that were made. You don’t have to become traumatised then.’ This is a shortcoming of our regular system; women turn to me because they didn’t feel involved in decision-making during their previous birth.”*

(Caseload midwife 2)

An obstetrician explains her view on the role of the care provider and the role of women. This obstetrician is able to accept women’s birth plans, even if she would have made different decisions if she was pregnant herself, because she believes that in the end, the woman needs to decide.

*“It’s about your view on your profession. A lot of people […] are educated—not explicitly—with the idea of ‘a patient has a problem and you need to fix it’. And when something happens, you decide which interventions you’re going to do. In my opinion, this idea is outdated. I don’t think all birth plans are sensible. But for me it is easier to accept these birth plans when I think that it’s not my decision, it’s not my body and it’s not my child.”*

(Obstetrician 1)

According to one midwife, the maternity care system relies too much on guidelines and the needs and preferences of women are not central.

*“Everyone says ‘the patient is central’. I question that. […] The protocol is central. And care providers constantly talk about how we practise woman-centred care. I actually think that in the Netherlands we practise midwife- and obstetrician-centred care. […] That is what we do, we think it’s in the patients’ interest, we do it with the best intentions. That’s the reason why change is so hard, because everyone thinks that this is the best way to do it.”*

(Caseload midwife 1)

## 4. Discussion

The aim of this qualitative study was to examine obstetricians’ and midwives’ experiences with the outpatient clinic “Maternity Care Outside the Guidelines” in Nijmegen, the Netherlands. All participants cared for women who requested less care than recommended. In-depth interviews with ten care providers yielded knowledge about ways to improve care for women who request less care than recommended. Four main themes were identified: (1) “Trusting mothers, childbirth and colleague”; (2) “A supportive communication style”; (3) “Continuity of carer”; (4) “Willingness to reconsider responsibility and risk”. One overarching theme was found, which was “Guaranteeing women’s autonomy”. We will now discuss these themes, the relationship between them and how these findings relate to the existing literature. 

### 4.1. Achieving Mutual Trust through a Supportive Communication Style

Care providers in this study explained how the designated clinic aims to (re)build and maintain trusting relationships by listening, conveying support and showing motivation and flexibility to explore alternative care options outside the guidelines. A meaningful dialogue is established by the clinic’s approach of “how can we help you to give birth according to your own values and preferences, while keeping things as safe as possible?”. Respecting a woman’s right to refuse (parts of) care is a way to achieve trust and to keep women engaged in care [25,26,27,28]. Besides good communication, participants also regard investing time and continuity of carer as essential in building a trusting relationship. Jenkinson et al. [4] and Perriman et al. [29] confirm this. Participants in our study, as well as in other studies stated that discussing risks and negotiating birth plans become easier when there is trust [16,27]. 

Midwives in this study were supportive of women’s birth wishes because they trust the natural course of birth and women’s instinctive knowledge. Accordingly, the women interviewed by Lee et al. [30] mentioned that they felt empowered by their midwives because their midwives expressed trust in them, their body and the natural birth process. The stories of these care providers show that women who initially distrusted the hospital, started to trust the hospital again, after they visited the designated clinic. None of the women cared for by the participants left the maternity care system and none of the participants reported negative interactions with their client. 

This is in contrast with the results of a multiple case study by Holten et al. [23]. It showed that women decided to leave the maternity care system because the interactions with their care providers left them no other choice. They experienced poor communication and inflexibility on the part of their care providers. During consultations, women were not asked about the reasoning behind their wishes and their preferences were overruled. This behaviour is described by Jenkinson et al. [4] as “Escalating intrusion”: manipulating women into consenting to recommended care and telling women that recommended care would be performed, with or without their consent. In summary, the women interviewed by Holten et al. [23] could not trust a care provider who refused to respect their autonomy and who tried to coerce them to consent to recommended care. The interactions between women and care providers in this current study strengthened relationships, whereas the interactions in the study by Holten et al. [23] were harmful to the development of (trusting) relationships. 

### 4.2. How Trust, Medicalisation, Freedom of Choice and Negative Choices Are Related

While pregnancy and birth have become more safe (according to biomedical standards)—due to our increased ability to detect and treat abnormalities—care providers seem to have become more fearful of adverse events. To minimise the risk of an adverse event, birth is continuously controlled and monitored [31,32,33,34]. Many articles have been published on why women request less care than recommended [1,2,4,7,10,11,15,23]. Some women decline recommended care because they wish to avoid being subjected to the routine interventions performed in hospital care. Throughout the literature it is described how women who seek physiological birth are hindered by a risk-averse system that is dominated by medicalisation and guidelines [1,7,11,15,16,35]. Other women experience a lack of choice in our current maternity care services [1,15,16,23,35]. Women deemed high-risk have limited choices regarding place of birth, birthing position, waterbirth or midwifery-led continuity of care during birth [16]. Women interviewed by Holten et al. [23] experienced a lack of choice when they presented their birth intentions to their care providers. Thompson [36] argues that this ‘choiceless choice’ is a result of the routinisation of obstetrical interventions, which excludes women from decision making about their own care. In other words, the question can be raised whether our existing biomedically focussed maternity care services are sufficiently meeting women’s emotional and social needs [5,7,15,16,35]. This gap between women’s needs and our current maternity care services could have a meaningful impact on women’s birth intentions and experiences. 

The wish to prevent a repeat of traumatic events encountered in a previous birth, is another major reason for women to decline recommended care [7,10,11,12,15,23]. For example, the majority of women included by Jackson et al. [7] perceived their previous birth experiences to be emotionally and physically unsafe, and as a result, traumatizing. Women who have had a traumatic birth experience have learnt that a hospital birth inside the system may be riskier for them than a birth outside the system [1,7,10,15,23]. If we continue to make women believe that the hospital is unsafe by not letting them make their own decisions during birth—even if we do not agree with those decisions—women are likely to keep making negative choices. 

### 4.3. Continuity of Carer

This study revealed that care providers regard relational continuity as essential for the development of trusting relationships. Continuity of carer has shown to be beneficial for both women and care providers [18,19,29,37]. Perriman et al. [29] examined women’s experiences with caseload midwifery. Women explained that they felt known and understood as an individual. This encouraged them to take the lead in decision making. 

Furthermore, negotiating a birth plan can become easier when women receive care from one care provider [16,27,38]. For nine out of ten participants in this study, their client’s motivation to decline recommended care was the desire for continuity of carer from their own community midwife. One could argue that some women with a high-risk pregnancy choose alternative care options, such as a caseload midwife, because they miss relational continuity in regular care [35]. 

### 4.4. Risk and Responsibility

Literature shows that care providers may feel vulnerable and find it emotionally difficult to accept a woman’s decision when this decision, in the professional’s perspective, may put her and/or her child at risk. Other care providers are apprehensive regarding liability issues in case of a bad outcome when the woman’s wishes are respected [3,4,13,30].

Respect and support for maternal autonomy is a core ethical principle, even when a woman’s decision involves placing herself and/or her foetus’ life at risk. It is the right of every patient to make informed and autonomous health care decisions, including refusing care or treatment. If a woman has decisional competence and is well-informed, then her refusal cannot be overruled [13,14,39]. Care providers cannot be held accountable for the consequences of a woman’s decision, provided certain conditions are met. It is the care provider’s duty to adequately inform women about why certain care or intervention options are recommended in her situation, the risks and benefits of the care that is recommended, alternatives to treatment and the risks and possible consequences of refusing recommended care. Documenting everything that is discussed during these conversations with the woman is essential: the woman’s refusal, her reason(s) for her refusal, the information that is given and the final care plan that is agreed on [14,20]. 

### 4.5. A Structured Approach

The dialogues at the clinic are aimed at exploring and clarifying each other’s perspective. The relevant guidelines are discussed in a respectful manner, with the aim to inform women about the potential consequences of different care options and to guide them in their decision-making process. Van der Garde et al. [17] described the clinic’s approach: “The clinic’s aim is to use a respectful and systematic multidisciplinary approach, in which women feel heard and are invited to explain their motivations for their birth plans, and by doing so arriving at a plan either compatible with or much closer to recommendations than the woman’s initial intentions in most cases, thereby preventing negative choices” [17]. Its aim is not only to protect mothers and their children, but also to take the emotional risk for the medical team into account [17]. After a few consultations at the clinic, a care plan is agreed on. This can involve a community midwife-led hospital birth, obstetrician-led care with fewer interventions than recommended or a home birth with a community midwife (Table S3). The care providers working at the clinic are not personally responsible for intra-partum care. This will be delivered by a woman’s community midwife, a hospital-based midwife and/or by the obstetrician on duty. On-duty care providers are not obliged to provide this care if they do not support the care plan. However, they are obliged to find someone else within the hospital staff or the midwifery practice who is willing to provide this care. When this happens within the hospital, one of the obstetricians who run the clinic is available for when the specific patient goes into labour. Similar to this approach are the approaches elaborated on within the literature, for instance, the PACT-framework and the maternity care plan described by Jenkinson et al. [40,41].

### 4.6. Autonomy 

All participants in this study acknowledge women’s right to make their own decisions, including the right to refuse care or treatment. This commitment to maternal autonomy is also observed by Jenkinson et al. [4], Symon et al. [28] and Wickham [42]. However, Kruske et al. [43] reported that care providers’ knowledge and understanding of maternal and foetal rights and legal accountability are deficient. 

They concluded that maternity care providers say they are committed to maternal autonomy, but do not translate this into practice. It seems that care providers only support women’s autonomy when the decision she makes lies within the care provider’s personal limits [4,23,43]. In our study, all obstetricians and midwives believe in the importance of a positive birth experience and understand why women may choose to decline certain care after a traumatic birth experience. However, for a substantial number of interviewed care providers, this does not mean that they are willing to agree to women’s birth intentions when those intentions are in conflict with their personal values. The point at which care providers are not willing to go along with a woman’s request anymore is identified by Jenkinson et al. [4] as the “line in the sand” and can be different for every care provider [4,25,40,44]. In other words, for some care providers, support for maternal autonomy has its boundaries. Jenkinson et al. [4] questions whether care providers are fully aware of how their own values impact women’s freedom of choice. 

### 4.7. Strengths and Limitations

There are several strengths to this study. The main strength of this study is that this is the first qualitative study of care providers’ experiences with care outside the guidelines in the Netherlands. A second strength is the interprofessional research team including authors with different backgrounds in maternity care which offers broad perspectives on the subject. In addition, the participants are a good representation of the Dutch maternity care system, including obstetricians, hospital- and community-based midwives and caseload midwives. There are also some limitations. First, the two obstetricians who run and manage the outpatient clinic, were necessarily included as participants, and are also co-authors of this article. This could influence the results and their interpretation. 

On the other hand, the role of the researchers as participants and authors may provide them with a better understanding of the perspectives and challenges of the other participants. This is similar to the data collection method used in ethnography, where the researchers actively engage in the activities of the participants. Second, other participants were aware that, when sharing their views and thoughts on the clinic, their colleagues read and analysed the data. This may have caused them to give socially desirable answers. 

### 4.8. Implications for Practice

The results of this study reveal how care providers in the designated clinic built mutual trust with women, and as a result, successfully negotiated birth plans. However, the ability to stand by women who request less care than recommended does not resolve the mismatch between women’s needs and our maternity care system. Guidelines are evidence based and have proven clinical benefit to women. This evidence should not impact patient choice, it should be seen from the personal perspective of the individual woman and support her in making her own decisions. If we give women trust and choices, then we can prevent women with a high-risk pregnancy from feeling forced to birth at home because they did not feel safe and listened to in the hospital. 

Setting up special outpatient clinics is not a long-term solution. A change in attitude towards true woman-centred care and Shared Decision Making is needed. Women should be asked what is important to them, and every woman should be able to decide on a care plan that she perceives as safe and empowering, even if this is not in line with advice from professionals, or the available evidence. 

One solution could be more interdisciplinary care plans and shared care between community midwives, hospital-based midwives and obstetricians. This would allow women to receive care from one known care provider throughout their pregnancy and birth, and enable women to have an (indicated) hospital birth—under the supervision of an obstetrician—with their own community midwife by their side. The approach used at the designated clinic in Nijmegen could also be helpful for maternity care providers in other contexts: it could make them feel more prepared and less vulnerable when a woman declines recommended care [3,8,26]. The psychological impact on care providers when poor outcomes occur must be taken into account, and healthcare institutions should formulate a plan on how to act when this affects medical teams and provide adequate support and (legal) protection for their employees.

### 4.9. Directions for Future Research

Further research could be aimed at supporting care providers in their communication with women throughout the decision-making process. Additionally, research is needed on how to give care providers a feeling of safety and confidence when working outside the guidelines. An analysis of cases of births outside the guidelines offers insight into the psychological impact on care providers when poor outcomes occur.

At a national level, more evidence is needed on how to make continuity of carer available for more women, how to adapt the maternity care system to realise that continuity and the potential cost implications of this.

## 5. Conclusions

Our study revealed how to prevent women from making negative birth choices that lead to births outside the system. Special outpatient clinics for care outside the guidelines are not a long-term solution. Women should be able to birth in the way that they perceive as safe and empowering. Maternity care systems should support women in making well-informed, autonomous choices within trustful care relationships, in order to prevent negative choices.

## Data Availability

Interview transcripts and audio recordings are stored in the Radboud University file folder for the minimum period of 10 years. Due to the privacy of participants, interview transcripts are not publicly available. Anonymised transcripts can be provided by the corresponding author upon request.

## Supplementary Materials

The following are available online at https://www.mdpi.com/article/10.3390/ijerph182111627/s1, Table S1: Characteristics of the participants (*n* = 10); Table S2: Topics addressed in the interviews; Table S3: Characteristics of the ten cases.
