# Supplementary material for: How to Make the Hospital an Option Again: Midwives’ and Obstetricians’ Experiences with a Designated Clinic for Women Who Request Different Care than Recommended in the Guidelines
Source: Int J Environ Res Public Health. 2021 Nov 5;18(21):11627. doi: 10.3390/ijerph182111627 (PMC8583448; doi:10.3390/ijerph182111627)
Supplement: Supplementary file 1 [file ijerph-18-11627-s001.zip › ijerph-1443302-supplementary.pdf]

Table S1. Characteristics of the participants (n=10).

Characteristic		Role in care process	The client's initial request
**Women, (n)**	7		
**Men, (n)**	3		
**Age (yrs)**			
**<30**	0		
**31-40**	5		
**41-50**	2		
**51-60**	3		
**>60**	0		
**Profession, (n)**			
**Community midwife**			
**1**		Referral to the clinic	Midwife-led VBAC
**2**		Referral to the clinic	Midwife-led VBAC
**3**		Referral to the clinic	Midwife-led VBAC
**Caseload midwife**			
**1**		Referral to the clinic	Midwife-led VBAC
**2**		Referral to the clinic	Midwife-led VBAC
**Hospital-based midwife**		Intrapartum care	Waterbirth VBAC
**Obstetrician (working within the clinic)**			
**Obs. 1**		Decision-making process and intrapartum care	Twin birth (vaginal), request: no vaginal examinations, no IV-access, No foetal monitoring during birth.
**Obs. 2**		Decision-making process and intrapartum care	Midwife-led VBAC

**Obstetrician (working with the clinic)**		
**Obs. 3**	Intrapartum care	Midwife-led VBAC
**Obs. 4**	Intrapartum care	Midwife-led VBAC
**Work experience (yrs)**		
**1-5**	1	
**6-10**	5	
**10-20**	1	
**>20**	3	

Table S2. Topics addressed in the interviews.

- **Experience with working outside the guidelines:**
  General introduction of the specific case
  The client's request(s) and care provider's thoughts, opinions and feelings about it
  Participants' reaction to the request(s)
  The initial medical recommendation
  The decision-making process at the clinic and the care provider's role in this
  The participant's agreement and satisfaction with this process
  Flexibility on both sides
  Discussion of medical advice, risks and interventions with client
  Role of the birth plan
  Relationship with the client
  Trust and how to obtain it
  How was the communication between care provider and client?
  Mutual expectations
  The collaboration with other professionals at the clinic
  How do you look back on this specific case?

- **Your opinion about the outpatient clinic**
  What are its strengths?
  What could be done better?

- **Values and vision on maternity care**
  Trust: what does trust mean to you and how do you achieve it?
  What is a good birth?
  Meaning of personal care
  How to tailor care?
  What is good communication? What is bad communication?

- **Concerns, barriers and fears**
- **How do you perceive risks?**
- **Guidelines**

- **Shared Decision Making**
  What do you think of Shared Decision Making?

Table S3. Characteristics of the ten cases.

**Participant: woman's medical indication**	**G/P**<br>**Age**<br>**Gestational age**	**The woman's request/refusal**	**The birth plan after negotiation**	**Outcomes**
**Obs. 1: twin pregnancy**		Vaginal birth: no vaginal examinations, no intravenous-access (IV-access), no continuous intrapartum foetal heart monitoring, no medication postpartum. Birth on All-Fours.	No intravenous-access (IV-access), no continuous electronic foetal monitoring.<br>No active management after twin one and postpartum: interventions only if complications arise<br>Ultrasound examination after birth of first twin<br>Birth on All-Fours.	
**Obs. 2: previous caesarean section**		Vaginal birth: midwife-led continuity of carer in the hospital	Hospital birth led by a community midwife<br>Shared Care between community midwife and obstetrician<br>Continuous electronic foetal monitoring	
**Obs. 3: previous caesarean section**		Vaginal birth: midwife-led continuity of carer in the hospital	Hospital birth led by a community midwife<br>Shared Care between community midwife and obstetrician<br>Intermittent foetal heart auscultation	

**Obs. 4: previous caesarean section**	Vaginal birth: midwife-led continuity of carer in the hospital<br>Interventions only if complications arise	Hospital birth led by a community midwife<br>Shared Care between community midwife and obstetrician<br>Interventions only if complications arise
**Community midwife 1: previous caesarean section**	Vaginal birth: midwife-led continuity of carer in the hospital<br>No continuous intrapartum foetal heart monitoring	Hospital birth led by a community midwife<br>Shared Care between community midwife and obstetrician<br>Intermittent foetal heart auscultation
**Community midwife 2: previous caesarean section**	Vaginal birth: midwife-led continuity of carer in the hospital	Hospital birth led by a community midwife<br>Shared Care between community midwife and obstetrician<br>Continuous electronic foetal monitoring
**Community midwife 3: previous caesarean section and previous post-partum haemorrhage**	Midwife-led homebirth<br>No vaginal examinations<br>No IV-access	Hospital birth led by a community midwife<br>Shared Care between community midwife and obstetrician<br>Intermittent foetal heart auscultation<br>No IV-access

**Caseload midwife 1: previous caesarean section**	Midwife-led homebirth	Hospital birth led by a community midwife<br>Shared Care between community midwife and obstetrician<br>Continuous electronic foetal monitoring
**Caseload midwife 2: previous caesarean section**	Vaginal birth: midwife-led continuity of carer in the hospital	Hospital birth led by a community midwife<br>Shared Care between community midwife and obstetrician<br>Continuous electronic foetal monitoring
**Hospital-based midwife 1: previous caesarean section**	Water birth<br>No vaginal examinations, no IV-access<br>A hands-off birth<br>No active management of labour	Obstetrician-led care in the hospital<br>VBAC, water birth<br>No vaginal examinations, no IV-access<br>Hands-off birth<br>Continuous electronic foetal monitoring<br>No active management of labour<br>Interventions only if complications arise
